# Transcriptomics of *Pseudomonas aeruginosa* PA14 upon deletion of the sigma factor RpoS

**DOI:** 10.1128/mra.00433-25

**Published:** 2025-06-10

**Authors:** Luis Mauricio Salazar-Garcia, Jose Manuel Villalobos-Escobedo

**Affiliations:** 1 Industrial Genomics Laboratory, FEMSA Biotechnology Center, School of Engineering and Sciences, Tecnológico de Monterrey 27746, Monterrey, Nuevo Leon, Mexico; 2 Integrative Biology Research Unit, The Institute for Obesity Research, Tecnológico de Monterrey 27746, Monterrey, Nuevo Leon, Mexico; The University of Arizona, Tucson, Arizona, USA

**Keywords:** transcriptomics, RpoS, gene regulation, *Pseudomonas*

## Abstract

We report RNA-seq data of *Pseudomonas aeruginosa* PA14 (UCBPP-PA14) and an isogenic *rpoS-STOP* mutant generated using CRISPR/Cas9 base editing. Transcriptomic profiling during exponential growth in rich medium highlights the regulatory influence of RpoS. These data enable exploration of RpoS-dependent responses in the virulent PA14 strain.

## ANNOUNCEMENT

*Pseudomonas aeruginosa* is a gram-negative opportunistic pathogen capable of infecting a wide range of hosts, including humans (1–3). Its pathogenicity is attributed to a broad array of secreted virulence factors and intrinsic antibiotic resistance mechanisms (4–7).

Sigma factors are key transcriptional regulators that enable global shifts in gene expression in response to environmental stimuli. Among them, RpoS (σ^S^) coordinates the general stress response and regulates genes involved in adaptation to nutrient limitation, oxidative stress, and the stationary phase (4, 8, 9). While the RpoS regulon has been extensively characterized in *P. aeruginosa* PAO1 (10, 11), strain-specific differences in genome content between PAO1 and the more virulent PA14 strain (12, 13) support the need for RpoS analysis in *P. aeruginosa* PA14.

In this work, we used *P. aeruginosa* PA14 as the parental strain to generate an *rpoS-STOP* mutant. The mutation was introduced using the base-editing plasmid pMBEC6 (14), which encodes an APOBEC1-nCas9-UGI fusion. We designed and cloned a specific single-guide RNA targeting *rpoS*, allowing the precise introduction of a premature *STOP* codon (Q157*) confirmed by Sanger sequencing. The mutated strain was plasmid cured and re-sequenced by Sanger technology to verify the successful introduction of the *STOP* codon prior to RNA extraction. Both *P. aeruginosa* PA14 and its rpoS-STOP derivative were grown in LB broth at 37°C with shaking at 200 rpm. Cultures were initiated from single colonies picked from LB agar plates. Each colony was used to generate a pre-culture in LB, which was subsequently used to inoculate three biological replicates. OD_600_ was monitored, and cells were harvested at mid-log phase (OD_600_ ≈ 2.2, ~8 h). RNA was extracted using the Jena Bioscience Total RNA Purification Kit (PP-210S). RNA purity and integrity were assessed using a Nanodrop spectrophotometer and agarose gel electrophoresis. All samples had an RNA Integrity Number greater than 8. The RNA-seq libraries were prepared using the Zymo-Seq RiboFree Total RNA Library Kit (Zymo Research, CA, USA) and sequenced using Illumina NovaSeq 6000 (2 × 150 bp paired end). On average, 30 million read pairs were generated per sample. For the analysis, default parameters were used for all software unless otherwise specified. Reads were trimmed with fastp (version 0.23.2) (15), and quality control metrics showed that over 95% of reads passed filtering. Alignment to the *P. aeruginosa* PA14 reference genome (NC_008463.1) was performed using HISAT2 (version 2.2.1) (16), and quantification was carried out using featureCounts (version 2.0.3) (17), revealing that an average of 93.7% of reads mapped to annotated coding sequences. Differential expression analysis was carried out using DESeq2 (version 1.40.1) (18) with an adjusted *P*-value < 0.05 and a log2 fold change >1 or <−1 were considered significantly upregulated or downregulated.

**Fig 1:**
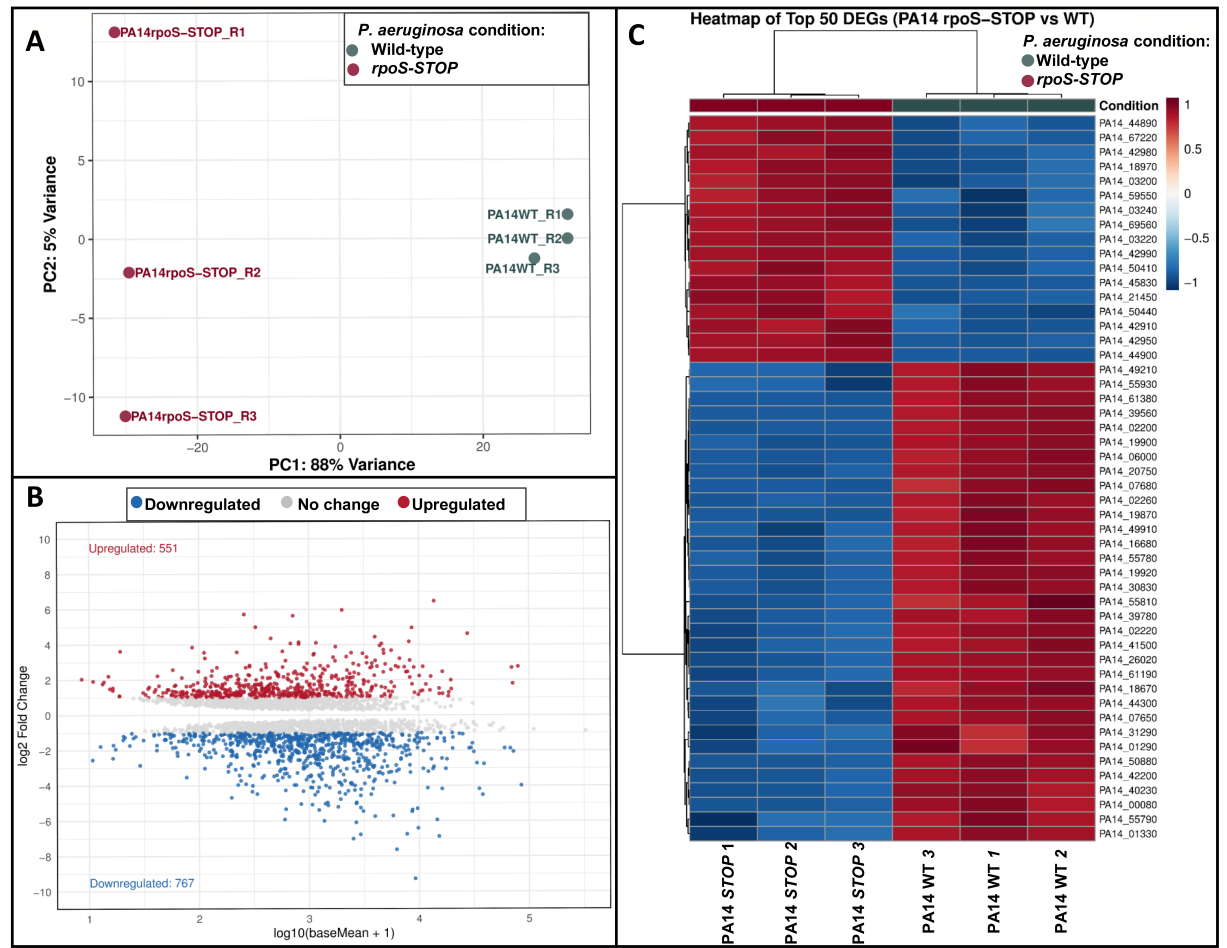
Transcriptomic analysis of *P. aeruginosa* PA14 wild-type and *rpoS-STOP* mutant strains. (**A**) PCA plot showing clear separation between wild-type (PA14_WT) and *rpoS-STOP* (PA14_rpoS-STOP) samples. PC1 accounts for 88% of the variance. (**B**) Volcano plot of differentially expressed genes (DEGs), with significantly upregulated (red, *n* = 551) and downregulated (blue, *n* = 767) genes in the *rpoS-STOP* mutant compared to the wild-type strain (adjusted *P*-value < 0.05). (**C**) Heatmap of the top 50 DEGs based on adjusted *P*-value, displaying log-transformed normalized expression averages across biological replicates. Red indicates higher expression, and blue indicates lower expression levels. PCA, differential expression analysis, and visualization of transcriptomic data were performed in RStudio (version 4.2.2) using the DESeq2, ggplot2, and pheatmap packages.

Principal component analysis (PCA) confirmed distinct transcriptional profiles between the wild-type and the *rpoS-STOP* mutant strains (Fig. 1A). The PCA, differential gene expression, and data visualization (volcano plot and heatmap) were performed in RStudio (version 4.2.2) using the DESeq2, ggplot2, and pheatmap packages. We observed 551 genes significantly upregulated and 767 downregulated in the mutant (Fig. 1B). These transcriptomics shifts (Fig. 1C) reflect the extensive regulatory scope of RpoS in PA14 and establish a framework for future studies.

## Data Availability

RNA-seq data from the *Pseudomonas aeruginosa* PA14 wild type and its rpoS-STOP mutant have been submitted to the NCBI Gene Expression Omnibus (GEO) under accession number GSE295781 (full data set); GSM8957634, GSM8957635, and GSM8957636 (PA14WT, replicates 1–3); and GSM8957637, GSM8957638, and GSM8957639 (PA14rpoS-STOP, replicates 1–3). Raw sequencing reads have been deposited in the Sequence Read Archive (SRA) under accession number SRP581755.
